# Combatting ventilator induced diaphragm dysfunction with human bone marrow mesenchymal stromal cell-derived extracellular vesicles

**DOI:** 10.1186/s13395-026-00433-6

**Published:** 2026-06-13

**Authors:** Ya Wen, Xiang Zhang, Yvette Hedström, Nicola Cacciani, Jonas Bergquist, Y. Ikeno, Oscar E. Simonson, Sergey Rodin, Karl Henrik Grinnemo, Lars Larsson

**Affiliations:** 1 Center for Molecular Medicine (CMM), Karolinska Institutet, Stockholm, Sweden; 2 Department of Molecular Medicine and Surgery, Karolinska Institutet, Bioclinicum, Stockholm, Sweden; 3 Department of Clinical Sciences, SLU, Uppsala, Sweden; 4 Analytical Chemistry and Neurochemistry, Department of Chemistry – Biomedical Center, Uppsala University, Uppsala, Sweden; 5 The Myalgic Encephalomyelitis/Chronic Fatigue Syndrome (ME/CFS) Collaborative Research Centre at Uppsala University, Uppsala, Sweden; 6 Barshop Institute for Longevity and Aging Studies, Department of Pathology, UTHSCSA, San Antonio, USA; 7 Geriatric Research Education and Clinical Centers (GRECC), Audie L. Murphy VA Hospital (STVHCS), San Antonio, TX 78229 USA; 8 Department of Surgical Sciences, Division of Cardiothoracic Surgery, Uppsala University, Uppsala University Hospital, Uppsala, Sweden; 9 Viron Molecular Medicine Institute, One Boston Place, 6th Floor, Boston, MA 02108 USA

## Abstract

**Background:**

Prolonged mechanical ventilation is closely associated with ventilator-induced lung injury (VILI) and ventilator-induced diaphragm dysfunction (VIDD). These two conditions occur in parallel and contribute to delayed weaning, prolonged intensive care unit (ICU) stay, and poor clinical outcomes. This study evaluated whether human BM-MSC-derived extracellular vesicles (EVs) can simultaneously alleviate lung and diaphragm abnormalities in a unique rat experimental ICU (ExICU) model.

**Methods:**

Rats were subjected to 5 days of controlled mechanical ventilation with or without a single intravenous EV dose. Outcomes included lung histopathology, diaphragm single-fiber contractile function, transcriptomics and metabolomics of diaphragm muscle, proteomics and metabolomics of lung tissue, and serial proteomics of bronchoalveolar lavage fluid (BALF).

**Results:**

Five days of mechanical ventilation in the ExICU model were accompanied by severe lung morphological damage and approximately 50% reductions in diaphragm fiber size and specific force. EV treatment was associated with parallel improvements in lung pathology and diaphragm function. Multi-omics revealed coordinated molecular disturbances across lung, BALF, and diaphragm after mechanical ventilation, the majority of which were reversed by EVs.

**Conclusion:**

Our findings demonstrate an association between lung injury and diaphragm dysfunction during prolonged mechanical ventilation. BM-MSC-derived EVs exert parallel protective effects on both organs and represent a promising intervention to reduce complications of mechanical ventilation in critically ill patients.

**Supplementary Information:**

The online version contains supplementary material available at 10.1186/s13395-026-00433-6.

## Background

Intensive care units (ICUs) have undergone significant development resulting in higher survival rates in the past 65 years, primarily due to improved understanding of underlying pathophysiology, improved medical technologies, progress in therapies, and the introduction of evidence-based medicine resulting in removal of harmful interventions. Mechanical ventilation is a lifesaving intervention frequently used in critically ill ICU patients, but it is associated with various complications such as the Ventilator Induced Lung Injury (VILI) and the Ventilator Induced Diaphragm Dysfunction (VIDD) with negative consequences for patient quality of life (QoL), morbidity, mortality and health care costs. Weaning from mechanical ventilation is time-consuming and additional problems during weaning are observed in 20–30% of these patients, resulting in prolonged intensive care support, and increased risk of secondary pulmonary complications and mortality. The mechanism underlying the delayed weaning from the ventilator is intrinsic to diaphragm muscle fibers, rather than related to alterations in lungs, thorax/abdominal compliance, or neural input [1, 2]. These patients account for ~ 30% of overall ICU costs or a staggering $64 billion in the US annually [3]. Despite the impact of these lifesaving interventions in modern critical care, underlying mechanisms are elusive, and there is currently no effective treatment for VILI and VIDD, other than supportive care and intensive rehabilitation.

In the general ICU population, assisted ventilation is the preferred mode of mechanical ventilation since it reduces, but does not eliminate, VILI compared with controlled mechanical ventilation (CMV). However, CMV must be used in pharmacologically paralyzed patients or when there is an insufficient central ventilatory drive such as in many neuro-ICU patients, or in COVID-19 patients ventilated in the prone position. We have in two previous clinical studies followed neuro-ICU patients exposed to CMV longitudinally for 9 and 12 days [4, 5], All these patients developed an acquired muscle dysfunction compared with 30% in the general ICU population where assisted ventilation is used to reduce VILI [4, 5]. Thus, the lung injury caused by mechanical ventilation may play an important pathophysiological role underlying VIDD. Recent metabolomic and proteomic results from our group demonstrate an inflammatory response in lung tissue in response to prolonged CMV in an experimental ICU (ExICU) model [6] and the release of cytotoxic and inflammatory factors in bronchoalveolar lavage fluid (BALF) [7]. These observations were supported by lung histopathology showing over-inflation of air space with disruption of the alveolar wall (emphysema-like), commonly associated with edema in the alveolar space and perivascular area.

VILI and VIDD represent iatrogenic consequences of life-saving long-term mechanical ventilation unrelated to the underlying clinical condition. It is hypothesized that VIDD is a consequence of VILI and the release of cytokines/cytotoxic factors from the highly vascularized lung tissue. Investigating underlying mechanisms and intervention strategies induced by long-term exposure to mechanical ventilation *per* se in the absence of confounding disease is unethical in clinical studies. Experimental models allowing long-term observation periods are therefore needed since the iatrogenic consequences of modern critical care are strongly associated with the duration of mechanical ventilation. Further, the condition associated with long-term mechanical ventilation represents a complex biological system which cannot be replicated in in vitro models. Our unique murine ExICU model allows long-term studies of mechanisms and interventions offering an unprecedented opportunity to unravel mechanisms and evaluate specific interventions.

Bone marrow mesenchymal stromal cells (BM-MSCs) have excellent proliferative capacity which has positioned them as leading candidates for cell-based therapies. However. concerns have been raised regarding ethical and safety concerns, adverse immune response, and capacity to migrate to damaged tissues [8]. The therapeutic effects of BM-MSCs are mediated via secretome derived extracellular vesicles (EVs) which have distinct properties of long-distance communication, superior stability, high transport efficiency to damaged tissue, and improved safety profile versus BM-MSCs [9], i.e., EVs cannot self-replicate reducing the risk of tumor formation or vascular occlusion compared with BM-MSCs. Thus, systemic administration of BM-MSC derived EVs during long-term mechanical ventilation offers a novel approach to combat the lung injury associated with mechanical ventilation, i.e., targeting a potential pathophysiological mechanism underlying VIDD.

## Materials & methods

### Animals, experimental ICU model, and EV treatment

All animal work was approved by the ethical committees at Uppsala University (SLU.ua.2024.4.1–2671) and Karolinska Institutet (Dnr 3783 − 2024). A total of 26 female 265–334 g Sprague Dawley rats were included in this study. In the experimental ICU model, all rats were females since urine production was one of the monitored parameters. Our overall goal has been to make our experimental ICU model minimally invasive and the urine bladder was accordingly catheterized via urethra which is difficult, but possible in female rats. In male rats, this is to our knowledge not possible without introducing significant trauma. When evaluating our clinical material of ICU patients (*n* = 313) with acquired myopathies we have not observed any significant gender-related differences.

Experimental rats were exposed to deep sedation, post-synaptic neuromuscular blockade and controlled mechanical ventilation for 5 days, i.e., 5-day control rats (D5, *n* = 10) and compared with 5-day ventilated rats treated with EVs derived from human BM-MSCs for 5 days (D5EV, *n* = 7). Sham-operated control rats were included for comparison (D0, *n* = 8). The initial body weights in rats exposed to 5 days immobilization and mechanical ventilation were 326 ± 17 g and 278 ± 15 g at the end of the observation period. In rats treated with EVs, body weight was 315 ± 16 g at the start and 286 ± 11 g after 5 days. The initial and 5-day bodyweights of the rat were treated with human BM-MSCs 303 and 286 g, respectively. The rat treated with BM-MSCs was not included in any statistical analyses.

All experimental animals were maintained in fluid and nutritional balance throughout the duration of the experimental procedures by introducing: (1) intra-arterial solution (0.6 ml/h) containing 21 ml H_2_O, 24 ml 0.5 N lactated Ringer, 0.84 g oxacillin Na, 0.65 mg α-cobrotoxin, 0.3 mg vitamin K (Synkavite), 20 meq K^+^ (as KCl); (2) an intra-venous solution (0.6 ml/h) containing 26 ml H_2_O, 16 ml 0.5 N lactated Ringer, 20% glucose (Baxter, Deerfield, IL, USA), 0.32 g oxacillin Na for the initial 24 h, then 8.5% Travasol amino acids (Baxter) and 20% Intralipid (Kabi, Uppsala, Sweden) were added subsequently to provide adequate nutrients [10–13]. Body temperature, peripheral perfusion and oxygen saturation (measured continuously with an infrared probe in a hind limb paw, MouseSTAT, Kent Scientific corp., Torrington, CT, USA) were monitored and maintained in the physiological range. The sham-operated controls were anesthetized with isoflurane, maintained in spontaneous breathing, received intravenous and intra-arterial solutions, and sacrificed within 2 h of the initial isoflurane anaesthesia and surgery.

During surgery or any possible irritating manipulation, the anaesthetic isoflurane level, i.e., the Minimum Alveolar Concentration (MAC) was > 1.5%, which maintains the following states: (1) the electroencephalogram (EEG) was synchronized and dominated by high-voltage slow-wave activity; (2) mean arterial pressure, 90–100 mmHg, heart rate maintained below 420 beats/min; and (3) no evident EEG, blood pressure or heart rate responses to surgical manipulation. Isoflurane was delivered into the inspiratory gas stream by a precision mass-flow controller. After the initial surgery, isoflurane was gradually lowered (over 1–2 days) and maintained at MAC < 0.5% during the remaining experimental period. Rats were ventilated through a coaxial tracheal cannula at 72 breaths/min with an inspiratory and expiratory ratio of 1:2 and a minute volume of 180–200 ml and gas concentrations of 40% O_2_, 56.5% N_2_, and 3% CO_2_, delivered by a precision (volume drift < 1%/ wk) volumetric respirator. Airway pressure was monitored continuously as well as end-tidal CO_2_ (EtCO_2_) and normocapnic condition maintained (EtCO_2_ = 37–45 mmHg) as well as normoxia (SpO_2_ > 90%). Intermittent hyperinflations (6 per hour at 19–20 cmH_2_O) over a constant positive end-expiratory pressure (PEEP = 1.5 cm H_2_O) were set to prevent atelectases. Post-synaptic neuromuscular blockade was induced on the first day (150 µg α-cobrotoxin) and maintained by continuous infusion (187 µg/day). Mechanical ventilation started after the neuromuscular blockade induction avoiding hypercapnia and hypoxemia. Experiments were terminated after 5 days. Female rats were preferred because easier urine bladder catheterization for diuresis monitoring. One dose (20 µl aliquot of BM-MSCs-derived extracellular vesicles (EV) with concentration of 2.4 × 10^10^ particles/ml mixed with 980 µl of ice cold saline immediately before the injection) was given intravenously immediately after the initial surgery. The diuresis was maintained above 1 ml/h. In no case did animals show any signs of infections or septicemia.

The inspiratory air is humidified to reduce the risk of mucous formation in the respiratory tract and collected in condensing traps in the expiratory tract, frozen and stored at -20 °C and referred to as bronchoaveolar lavage (BAL) fluid.

BM-MSCs were purchased from American Type Culture Collection (ATCC) and cultured on laminin-521 coated plates in low glucose DMEM medium supplemented with 10% fetal bovine serum and Penicillin-Streptomycin. Laminin-521 was purchased from Biolamina Ab, Sweden and the cell culture plates were coated according to the manufacturer’s instructions. All the cultures were performed at humidified incubators at 37 °C, 5% CO_2_.

For passaging, the cells were washed twice with PBS and exposed to TrypLE Express enzyme for 8 min at 37 °C, 5% CO_2_. After that, the cells were gently detached from the surface using a scraper, pipetted to dissociate into single-cell suspension into 15 ml conical tube, and three volumes of the complete medium was added. Then, the cells were centrifuged at 280 g for 5 min, the pellet was resuspended in the complete medium and the cells replated at the concentration of 7 Kcells/cm^2^.

For production of EVs, BM-MSCs were cultured until approximately 80% confluency and carefully washed twice with PBS. After that, serum-free Opti-MEM medium was added and the cells were incubated for 48 h at 37 °C, 5% CO_2_. Then, the conditioned medium was collected and centrifuged first time for 5 min at 700 g to remove living cells keeping the supernatant and second time for 10 min at 2000 g to remove cellular debris. The cells were cultured for 24 h in the complete culturing medium and, after that, another production round was made as described above.

For isolation of EVs, the conditioned medium was passed through 0.22 μm filter and centrifuged for 1 h at 110 000 g. The pellet was resuspended in PBS, centrifuged for one hour at 110 000 g and resuspended in a small volume of PBS. After that, the EV preparations were sterilized by passing through a 0.22 μm filter and characterized using NanoSight to define concentration of particles transmission electron microscopy (TEM) and Western-Blot to assess expression of specific markers of EVs (Suppplementary figure). EV characterization included assessment of canonical EV markers and negative controls for cellular contamination to validate purity. Then, the EV preparations were diluted with PBS and freezing buffer described here (DOI: 10.1002/jev2.12238) was added to achieve final concentration of 2.4 × 10^10^ particles/ml. Finally, aliquots containing 20 µl of EVs were frozen and stored at -80 °C until use.

Animals were euthanized under deep isoflurane anaesthesia by removal of the heart. Immediately after rats had been euthanized, lungs and diaphragm muscles were gently dissected. One mid-costal diaphragm was immediately snap frozen in liquid propane chilled by liquid nitrogen and stored at -140 °C until further analysis and the other prepared for single muscle fiber contractile measurements (see below). One lung was formalin fixed for histopathology and the other lung was snap frozen for omics analyses.

### Transmission Electron Microscopy (TEM)

A 5 µl drop of the sample was placed on a formvar and carbon coated 200-mesh copper grid. The excess solution was removed by blotting with filter paper. The sample was then directly contrasted with 2% uranyl acetate. Excess of uranyl acetate was removed by blotting on filter paper. The contrasting step was repeated twice.

Dried grids were examined by Tecnai™ G2 Spirit BioTwin transmission electron microscope (Thermo Fisher/FEI) at 80 kV with an ORIUS SC200 CCD camera and Gatan Digital Micrograph software (both from Gatan Inc.).

### Lung histopathology

Lung samples were fixed in 10% formalin and embedded in paraffin. Five-micron thick sections were placed on glass slides and subsequently stained with hematoxylin-eosin (H&E) for histopathological evaluation. Severity of alveolar alveolar ectasia (emphysema like lesion that has disruption of alveolar wall); alveolar inflammation, and alveolar edema were scored based on the severity of lesions: Alveolar Ectasia: Grade 0: none; 1 < 25% of area; 2: 25–50%; 3: 50–75%; 4: >75%; Alveolar Edema: Grade 0: none; 1 < 25% of area; 2: 25–50%; 3: 50–75%; 4: >75%; Alveolar Inflammation: Grade 0: none; 1 < 25% of area; 2: 25–50%; 3: 50–75%; 4: >75%.

### Muscle tissue, membrane permeabilization and single muscle fiber contractile measurements

At the end of the required duration of exposure to the ICU condition, one mid-costal diaphragm muscle was quickly frozen in liquid propane cooled by liquid nitrogen and stored at -140 °C for subsequent RNAseq-based transcriptomics, proteomics and metabolomics analyses. The other mid-costal diaphragm was placed in relaxing solution at 4 °C and bundles of ~ 50 fibres were dissected free and tied with surgical silk to glass capillary tubes at slightly stretched lengths. The bundles were then treated with skinning solution (relaxing solution containing glycerol; 50:50 v/v) for 24 h at 4 °C, after which they were transferred to -20 °C. Within 1–2 weeks, the muscle bundles were treated with a cryo-protectant and snap frozen in liquid nitrogen-chilled propane and stored at -140 °C for long term storage [12, 14].

On the day of contractile measurements, bundles were transferred to a 2.0 M sucrose solution and subsequently incubated in solutions of decreasing sucrose concentrations (1.5–0.5 M) for 30 min each and finally kept in a skinning solution at − 20 °C. A single fiber was removed from the muscle bundle and was placed between two connectors. One connector leads to a force transducer (model 400 A, Aurora Scientific), and the other to a lever arm system (model 308B, Aurora Scientific). The two extremities of the fiber were attached to the connectors as previously described [15]. The apparatus was mounted on the stage of an inverted microscope (model IX70; Olympus). While the fiber segments were in relaxing solution, the sarcomere length was set to 2.65–2.75 μm by adjusting the overall segment length and controlled during the experiment using a high-speed video analysis system (model 901 A HVSL, Aurora Scientific). The diameter of the fiber segment between the connectors was measured through the microscope at a magnification of ×320 with an image analysis system prior to the mechanical experiments. Fiber depth was measured by recording the vertical displacement of the microscope nosepiece while focusing on the top and bottom surfaces of the fiber [12, 13, 16]. The focusing control of the microscope was used as a micrometer. Fiber CSA was calculated from the diameter and depth, assuming an elliptical circumference, and was corrected for the 20% swelling that is known to occur during skinning [15]. Diameter and depth were measured at three different locations along the length of each fiber and the mean was considered representative of cell dimensions.

For the mechanical recording, relaxing and activating solutions contained (in mM) 4 Mg-ATP, 1 free Mg^2+^, 20 imidazole, 7 EGTA, 14.5 creatine phosphate, and KCl to adjust the ionic strength to 180 mM and pH 7.0. The concentrations of free Ca^2+^ were 10^− 9^ M (relaxing solution) and 10^− 4.5^ M (activating solution), expressed as pCa^2+^ (i.e., -log [Ca^2+^]). Apparent stability constants for Ca^2+^-EGTA were corrected for temperature (15 °C) and ionic strength (180 mM). The computer program from Fabiato [17] was used to calculate the concentration of each metal, ligand, and metal-ligand complex. Immediately preceding each activation, the fiber was immersed for 10–20 s in a solution with a reduced Ca^2+^-EGTA buffering capacity. This solution is identical to the relaxing solution except that the EGTA concentration is reduced to 0.5 mM, which results in a more rapid attainment of steady force during subsequent activation.

Force was measured by the slack-test procedure [18]. This was calculated as the difference between the maximal steady-state isometric force in activating solution and the resting force measured in the same segment while in the relaxing solution. Maximal force production was normalized to CSA (specific force, absolute force (P_0_)/CSA). For contractile measurements, strict acceptance criteria were applied. First, the sarcomere length was checked during the experiments, using a high-speed video analysis system (model 901 A HVSL, Aurora Scientific). A muscle fiber was accepted and included in the analyses: (i) if the sarcomere length of a single muscle fiber changed < 0.10 μm between relaxation and maximum activation, (ii) if maximal force changed < 10% from first to seventh activation [15]. The average single diaphragm muscle fiber CSA and specific force for each animal was used in the statistical analyses. Contractile recordings were excluded from one rat since resting tensions were unacceptable high and sarcomere pattern was irregular (six rats were included in the D0, D5 and D5EV groups).

### Sample collections and preparations for omics analyses

Diaphragm and lung tissues collected for omics analyses were snap frozen in liquid nitrogen and stored at -140 °C until RNAseq-based transcriptomics, LC-MS/MS-based proteomics and metabolomics analyses. BAL fluid samples were analyzed at day 1, 3, and 5, and centrifuged at 2000 × g for 5 min at 4 °C to remove cell debris, transferred into new 1.5 ml Eppendorf tubes, snap frozen in liquid nitrogen, and stored at -140 °C until LC-MS/MS-based proteomics [6, 7].

For transcriptomic analyses, differential expression analyses were performed using the edgeR package. Differentially expressed genes (DEGs) were defined as those with |logFC| > 1 and *p* < 0.05, i.e., a widely used and well-established criterion for DEG identification. For proteomic and metabolomic data, differential analyses were conducted using a generalized linear model (GLM) in R, and statistical significance were defined as *p* < 0.05 and FDR < 0.1. In addition, pathway enrichment analyses were performed using the clusterProfiler package, and pathways with *p* < 0.05 and FDR < 0.1 were considered statistically significant. Furthermore, granulator performs single-cell deconvolution by using reference gene expression signatures derived from scRNA-seq data to estimate cell-type composition in bulk RNAseq samples through a variety of linear modeling and machine-learning algorithms.

Each group (D0, D5, and D5EV) included six biological replicates. The main comparisons in this study were D5 vs. D0 and D5EV vs. D5 consistent with the experimental design and the biological questions addressing: (1) Effects of the exposure to the 5-day ICU-condition (D5 vs. D0). (2) Evaluate the therapeutic effects of the EV treatment on the pathological changes induced by the ICU condition (D5EV vs. D5).

### RNAseq-based transcriptomics of diaphram tissues

Total RNA was extracted from diaphragm muscles using RNeasy^®^ Fibrous tissue mini kit (Qiagen, Inc., Valencia, CA, USA) according to the manufacturer’s instruction. After quality control, a total amount of 1 µg RNA per sample was used as input material for library preparations and barcoded using NEB-Next Ultra™ RNA Library Prep Kit for Illumina^®^ (NEB, USA). The library preparations were subject to sequencing on an Illumina platform (Nova Seq 6000)(6).

### Cellular deconvolution

The estimation of cell type composition in diaphragm tissue from D0, D5, and D5EV groups was accomplished by integrating our bulk RNA-seq data with publicly available single-cell information. This integrated approach enabled comprehensive characterization of cellular heterogeneity within the experimental groups.

First, we constructed a single-cell reference atlas using publicly available single-cell RNA-seq (scRNA-seq) datasets of mouse muscle samples [19]. These datasets were processed through a standardized workflow implemented in the Seurat package (version 5), which included rigorous quality control, normalization, data scaling, integration, principal component analysis (PCA), clustering, t-distributed stochastic neighbor embedding (t-SNE) for dimensional reduction, and cluster visualization [20]. Cell type annotation was performed by referencing the CellMarker 2.0 database and previously published studies, ensuring accurate identification of distinct cellular populations within the muscle tissue [21, 22].

Subsequently, we inferred the cell type composition of diaphragm tissue from each experimental group (D0, D5, and D5EV) through cellular deconvolution analysis of our bulk RNA-seq data using both granulator [23] and MuSiC2 package [24]. This complementary approach modeled gene expression levels as weighted sums of cell-type specific expression profiles, providing robust estimation of cellular proportions. The dual-package analysis not only validated the consistency of deconvolution results but also identified top-ranked cell type marker genes that were differentially expressed among sham-operated group (D0) and mechanical ventilation groups with (D5EV) versus without (D5) EV treatment, offering insights into the cellular mechanisms underlying the observed therapeutic effects.

### LC-MS/MS-based proteomics of lung tissues and BAL fluid samples

Proteins from lung tissue and BAL fluid samples were lysed in T-PER lysis buffer (Thermo Fisher Scientific) with complete protease inhibitors (Sigma-Aldrich) and diluted to 1.0 mg/mL. The total protein concentration in the samples was measured using the Bradford Protein Assay with bovine serum albumin (BSA) as a standard. Aliquots corresponding to 20 µg of proteins from each of the lung lysate and 10 µg of proteins from each of the pre-treated BAL fluid sample were taken out for digestion. The proteins were reduced, alkylated, digested by trypsin. The collected peptide filtrate was vacuum centrifuged to dryness using a SpeedVac system. Thereafter the samples were dried and resolved in 0.1% FA (formic acid) and the resulting peptides were separated with Easy nanoLC in reversed-phase on a C18-column with 90 min gradient and electrosprayed on-line to a QEx Plus-Orbitrap mass spectrometer (Thermo Finnigan) following a standard operation protocol [25]. Tandem mass spectrometry was performed applying HCD and fed into differential proteomics analyses.

### LC-MS/MS-based metabolomics of diaphragm and lung tissues

Lung and diaphragm metabolomics were performed by Metabolon (Durham, North Carolina). Metabolite extractions were prepared using the automated MicroLab STAR system from Hamilton Company. Several recovery standards were added prior to the first step in the extraction process for quality control purposes. After removing proteins and organic solvent, the resulting extract was stored overnight in liquid nitrogen before subject to untargeted metabolomics profiling using ultra-high performance liquid chromatography-tandem mass spectroscopy (UPLC-MS/MS) and fed into metabolomics analyses [6].

### Identification of Differentially Expression Genes (DEGs) in diaphragm

For transcriptomics data, the paired-end raw reads underwent various procedures, including quality control, alignment to Rattus norvegicus Rnor_6.0 reference genome, and counting using the the “nf-core/rnaseq” pipeline. A cutoff of p-value < 0.05 and absolute fold change > 2 was used in the identification of differentially expressed genes (DEGs) after 5-day ICU condition (comparison “D5 vs D0”) and EV treatment (comparison “D5EV vs D5”).

### Identification of Differentially Expressed Proteins (DEPs) in lung and BAL

Database search was performed using the Sequest algorithm, embedded in Proteome Discoverer 1.4 (Thermo Fisher Scientific) against database Rattus norvegicus downloaded from Uniprot in December 2022. The search parameters were: Enzyme: Trypsin. Fixed modification was Carbamidomethyl (C), and variable modifications were Oxidation (M), Deamidated (NQ). The search criteria for protein identification were set to at least two matching peptides.

The protein score (the higher the better), the sum of the scores of the individual peptides generated by the proteomics platform, was subject to differential analysis using a generalized linear regression model. For each spectrum and sequence, the Proteome Discoverer application uses only the highest scoring peptide. Differentially expressed proteins (DEPs) were identified when p-value was < 0.05.

### Identification of Differential Metabolites (DMs) in lung and diaphragm

Raw peak area data, generated by the metabolomics platform (Metabolon, Durham, North Carolina, United States), of all samples were subject to quality control. Initially, raw peak area data were subject to batch-normalization to remove batch effect. For each metabolite, the raw values in the experimental samples were divided by the median of those samples in each instrument batch, giving each batch and thus the metabolite a median of one. For each metabolite, the missing values were imputed as the minimum value across all batches in the median scaled data. Subsequently, the batch-normalized-inputed data were log-transformed, displayed a normal distribution and was used in subsequent statistical analyses. Each metabolite was annotated by Metabolon, including classification and pathways. One lung sample obtained after being exposed to 5-day ICU condition was abnormal according to analysis from Metabolon technician and was removed from subsequent analyses.

After quality control, differential analysis was performed by general linear model. Metabolites with p-value < 0.05 and FDR < 0.1 were assigned as differential metabolites (DMs). All differential metabolites in each tissue were subject to hierarchical clustering analysis by heatmap R package, respectively.

### Functional enrichment analysis of DEGs, DEPs and DMs

DEGs and DEPs from each comparison were subject to over-represented enrichment analysis by ClusterProfiler. Functional enrichment analysis was performed using the GO and KEGG database for DEGs and DEPs, whereas analysis for DMs were conducted using the proprietary pathway database provided by Metabolon. Terms and pathways were considered as significantly enriched if *p* < 0.05 and FDR < 0.1. The enrichment results were compared among all comparisons to reveal similarities and differences. Z-score was used to indicate the downregulated (inhibited) or upregulated (activated) status of the enriched GO terms and KEGG pathways.

$$\:Zscore=(up-down)/\sqrt{count}$$

*Count* is the number of DEGs and DEPs assigned to an enriched term, where *up* and *down* are the number of assigned DEGs and DEPs increased or decreased, respectively.

### Protein-Protein Interaction (PPI) network analysis

Diaphragm DEGs, lung DEPs and BAL fluid DEPs from each comparison were subject to Protein-protein interaction (PPI) network analyses. PPI network analysis is based on database STRING via data mining of previous publications and experimental reports. Each link in the PPI network was evidence-based. The node (DEG) with highest network degree (based on the number of betweenness or association with other nodes) was considered as the network hubs with essential biological significance. The network was visualized by Cytoscape (version 3.82).

### Statistical analysis

All statistical analyses were performed in R statistical computing software version 4.1. Specifically, differential expression analyses were performed by edgeR for RNAseq, and by general linear regression for proteomics and metabolomics, followed by functional enrichment analysis (ClusterProfiler). *P* < 0.05 indicates a statistical significance. Based on prior studies, cohorts of *n* = 5 rats/grp per timepoint provide 80% power for a fold-change of 2 with 5% error by Student’s t-tests. Recurrence assays were increased to *n* = 6 rats/grp.

## Results

### Diaphragm muscle fiber size and specific force in EV treated and untreated animals exposed to 5 days mechanical ventilation

**Fig. 1:**
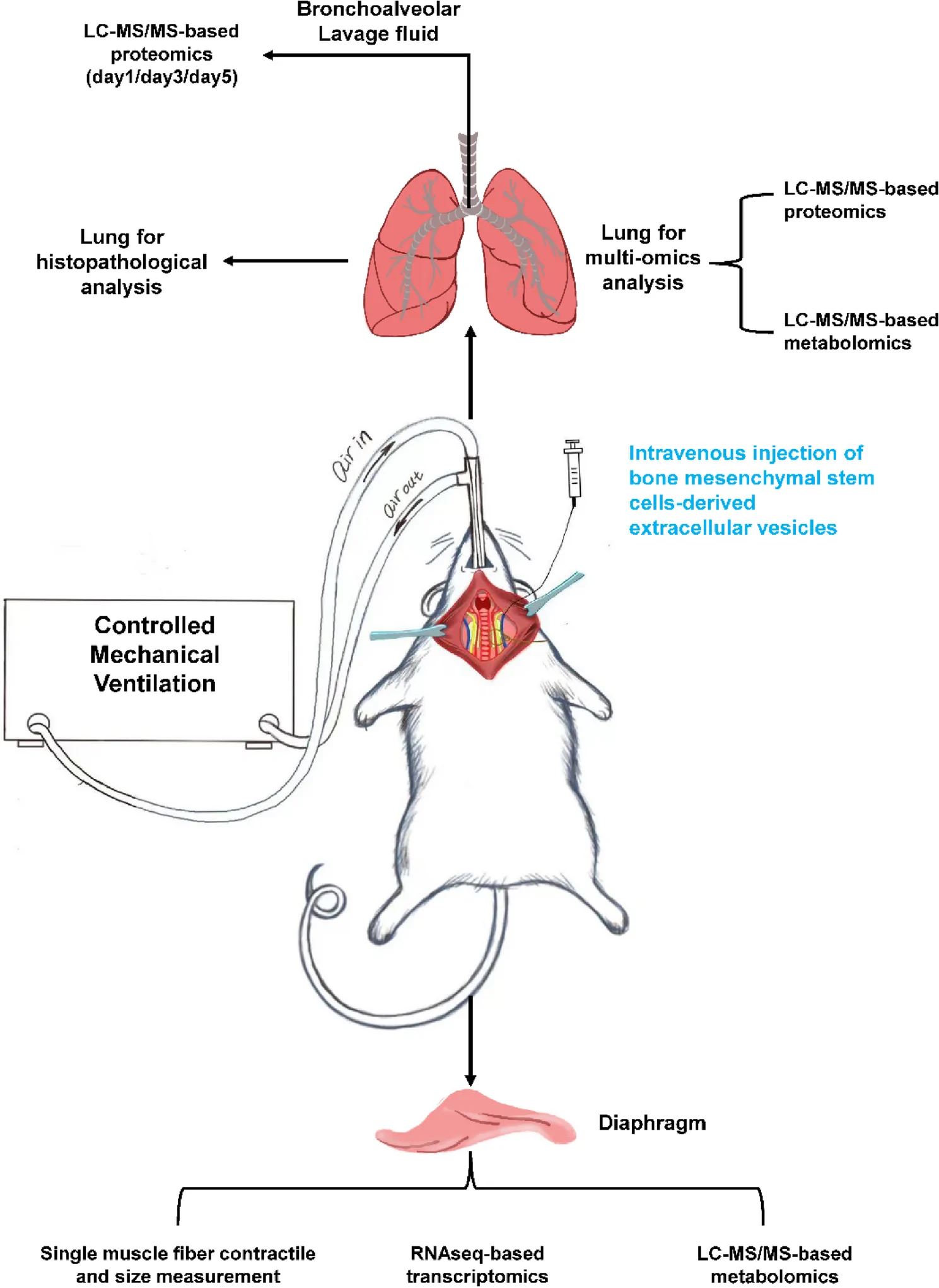
Schematic of experimental design for 5-day ExICU rat with EV intervention. D0: sham-operated rats (*n* = 8); D5: ExICU rats (*n* = 10); D5EV: ExICU rats with EV intervention (*n* = 7)

A schematic illustration of the experimental set-up is shown in Fig. 1. One successful 5-day experiment was performed in a rat treated with human BM-MSCs, but followed by a series of experiments where rats died within a couple of hours and interpreted to be due to adverse effects to the human BM-MSCs (hypoxia, hemodynamic instability, increased airway pressure without any signs of mucous plugs in the airways). The effects of BM-MSCs are, at least in part, mediated by biologically active EVs released from BM-MSCs. In following experiments, EVs derived from human BM-MSCs were therefore given systemically without showing adverse effects.

**Fig. 2:**
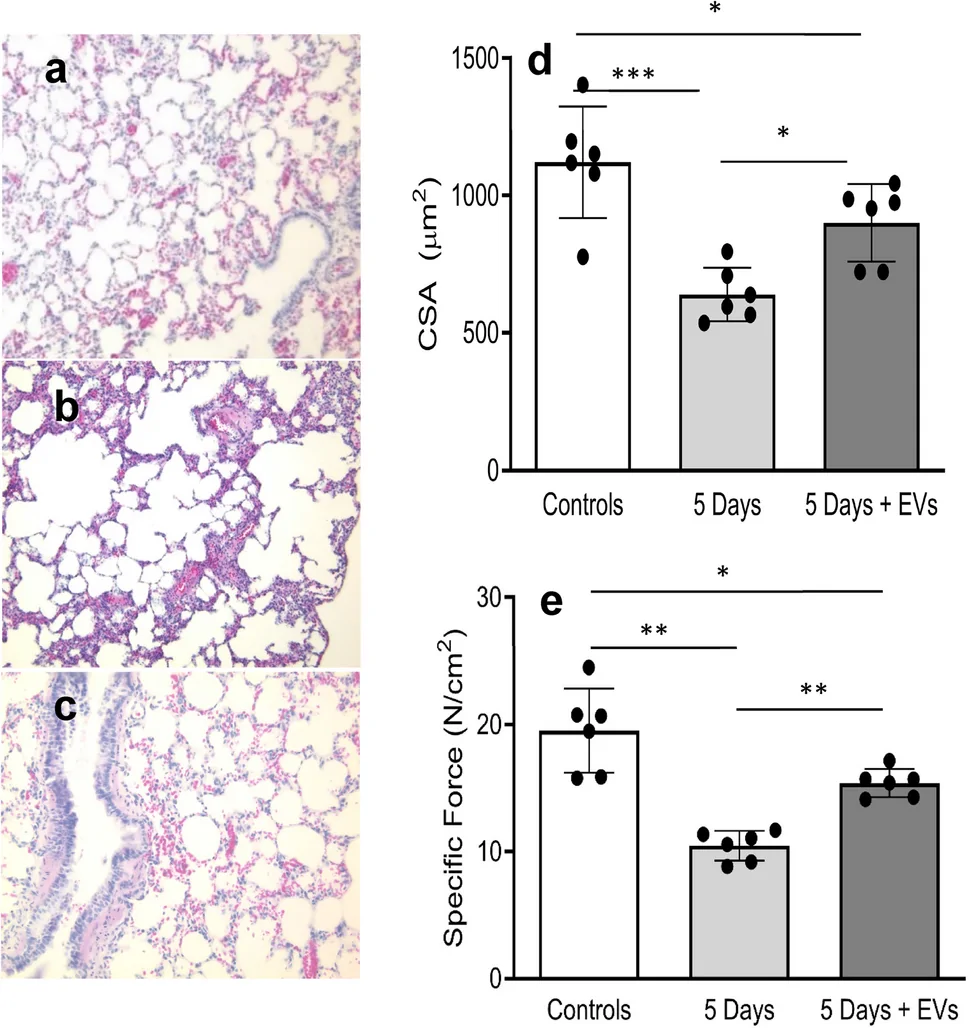
Lung histology and diaphragm muscle fiber structure and function. Representative histological hematoxylin-stained lung sections from (**a**) control, (**b**) 5 days mechanically ventilated, (**c**) 5 day mechanically ventilated EV treated rats, (**d**) single diaphragm muscle fiber cross-sectional area (CSA) and (**e**) maximum force normalized to fiber CSA (Specific force)

Single muscle fiber contractile recordings were performed in 18 rats, i.e., six rats in each of the D0, D5 and D5EV groups. A total of 267 diaphragm fibers (an average of 15 fibers per animal) met the acceptance criteria and were included in the study. Five days exposure to the ICU condition, i.e., immobilization and mechanical ventilation, had a strong negative effect on diaphragm muscle fiber size and function. Both diaphragm single muscle fiber cross-sectional area (CSA) and maximum absolute force normalized to muscle fiber CSA (specific force) were almost ~ 50% (*p* < 0.001) lower in rats exposed to the ICU condition for five days than in controls (Fig. 2). Thus, the decline in single muscle fiber force by far exceeded the decline in fiber size. The treatment with EVs improved both the CSA (*p* < 0.05) and the specific force (*p* < 0.001) compared with untreated animals (the average CSA and specific force for each rat was used in the statistical analyses between control and 5-day mechanically ventilated rats with and without EV treatment). The one rat treated with human BM-MSCs was within the same range for both diaphragm muscle fiber CSA (661 µm^2^, range 545–963 µm^2^) and specific force (16.6 N/cm^2^, range 8.5–20.8 N/cm^2^) as rats treated with EVs derived from human BM-MSCs (not included in statistical analyses).

### Lung morphology in EV treated and untreated animals exposed to 5 days mechanical ventilation

Histopathological evaluation of lungs showed increased alveolar pathology (edema, estasia, and inflammation) in rats exposed to the 5-day ICU condition. EV treatment reduced alveolar pathology with a significant (*p* < 0.018) decrease in alveolar estasia (emphysema like with disruption of aveolar wall) and similar trends were observed in alveolar inflammation and edema but failed to reach statistical significance (Fig. 2).

### Lung proteomics signatures in EV treated and untreated animals exposed to 5 days mechanical ventilation

**Fig. 3:**
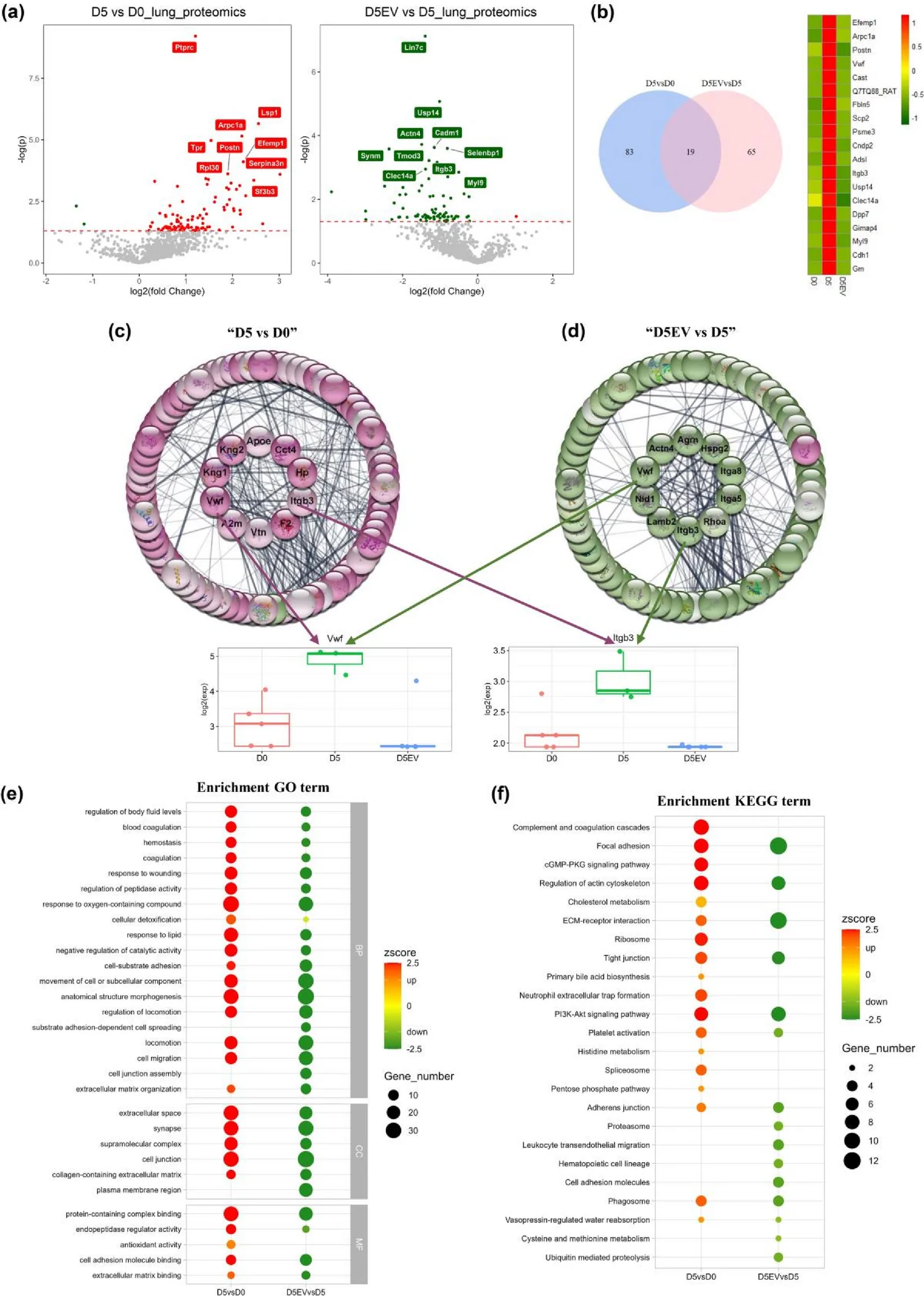
Proteomics reveals a restoring effect of EV treatment on molecular disarrangements associated with ventilator-induced lung injury (VILI). (**a**) Volcano plots of differentially expressed proteins (DEPs) in lung tissues of comparison “D5 vs D0” and “D5EV vs D5”; (**b**) the overlapped DEPs between comparisons “D5 vs D0” and “D5EV vs D5”; Hub DEPs in the protein-protein interaction network of comparison “D5 vs D0” (**c**) and “D5EV vs D5” (**d**). Comparative demonstration of enriched GO term (**e**) and KEGG term (**f**). D0: sham-operated rats (*n* = 5); D5: rats exposed to 5-day ICU condition (*n* = 4); D5EV: rats exposed to 5-day ICU condition and EV treatment (*n* = 5)

Differential analysis between ExICU rats and sham-operated controls (comparison “D5 vs D0”) identified a total of 102 differentially expressed proteins (DEPs) in lung tissues, whereas differential analysis between ExICU rats with and without EV treatment (“D5EV vs D5”) identified a total of 84 DEPs in lung tissues (Fig. 3a). Among which, 19 DEPs overlapped between comparisons “D5 vs. D0” and “D5EV vs. D5”, and all were with opposite direction of alteration, suggesting a restoring effect of EV treatment (Fig. 3b). Protein-protein interaction (PPI) networks were constructed by lung DEPs from the comparison “D5 vs. D0” (Fig. 3c) and “D5EV vs. D5” (Fig. 3d), respectively. The PPI network links were evidence-based. The node (DEP) with highest network degree (based on the number of betweenness or association with other nodes) was considered as the network hubs with essential biological significances. Vwf and Itgb3 were the overlapped hub DEPs, which were significantly upregulated in response to 5-day ICU condition and restored by EV treatment (Fig. 3c-d).

The enriched GO and KEGG terms from each comparison were selected for comparison. The z-score was employed to predict the upregulated/activated or downregulated/inhibited status of the enriched terms. A stronger z-score reflects a more significant up- or down-regulation. In the GO enrichment analyses, biological processes (BP) and cellular components (CC) related to injury, wound healing, and immune responses were significantly activated after the 5-day ICU condition, as reflected by the upregulation, or activated status of (positive z-scores) the GOBP terms “blood coagulation”, “response to wounding”, “anatomical structure morphogenesis”, “cell migration”, “extracellular matrix organization” (Fig. 3e), and KEGG terms “Focal adhesion”, “Platelet activation”, “Phagosome”, “PI3K-Akt signaling pathway”. These dysregulated biological processes and signaling pathways were partially restored by EV treatment (Fig. 3f).

### Lung metabolomics signatures in EV treated and untreated animals exposed to 5 days mechanical ventilation

**Fig. 4:**
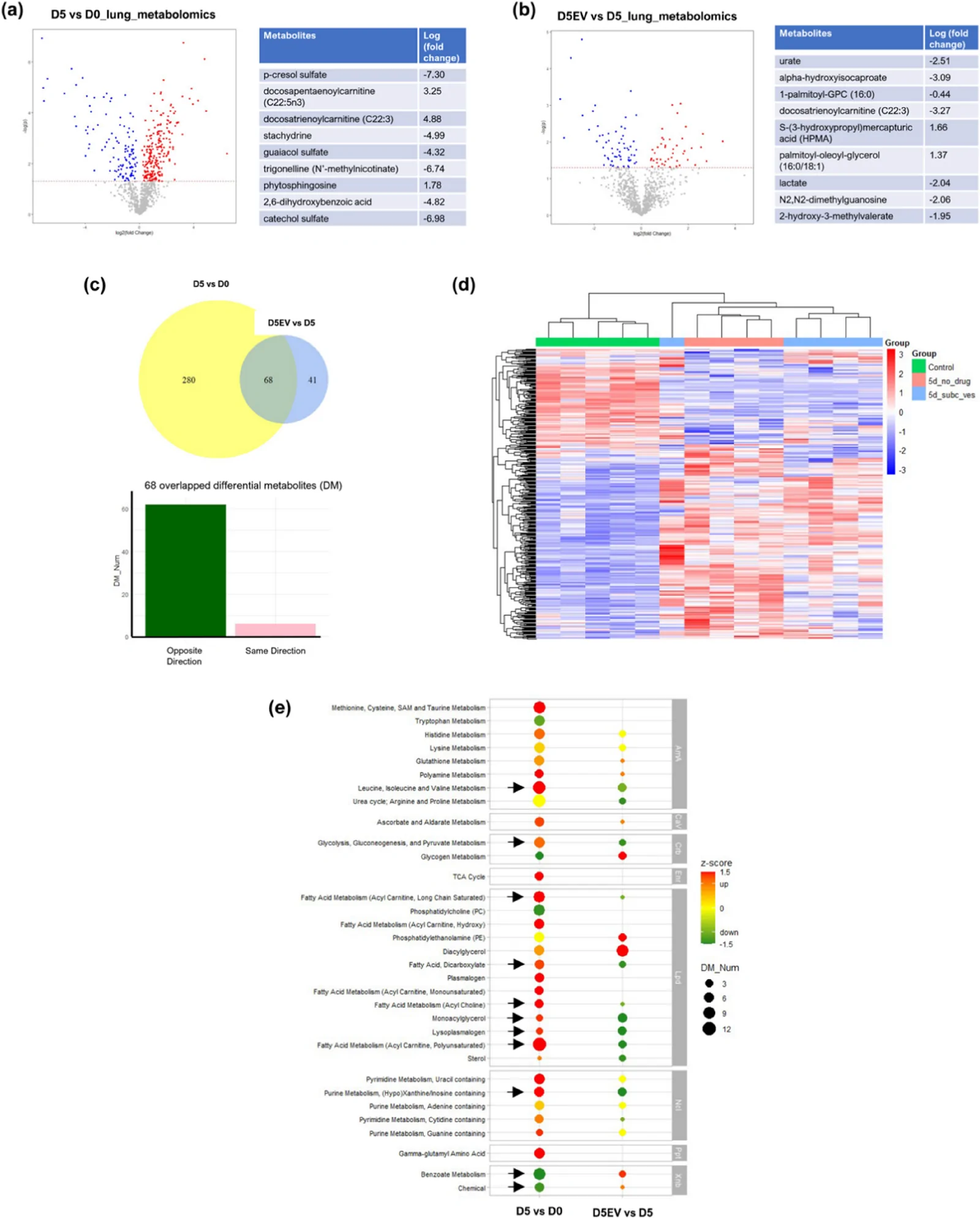
Metabolomics reveal the restoring effects of EV treatment on lung tissue. Differential metabolites (DMs) identified in “D5 vs D0” (**a**) and “D5EV vs D5” (**b**); (**c**) The overlapped DMs between comparisons “D5 vs D0” and “D5EV vs D5” and percentage of DMs with restoring effect of EV treatment; (**d**) Hierarchical clustering analysis of DMs in lung tissue of all groups; (**e**) Comparative demonstration of enriched metabolic pathways. D0: sham-operated rats (*n* = 5); D5: rats exposed to 5-day ICU condition (*n* = 4); D5EV: rats exposed to 5-day ICU condition and EV treatment (*n* = 5)

Differential analysis between ExICU rats and sham-operated controls (comparison “D5 vs D0”) identified a total of 348 differential metabolites (DMs) in lung tissues (Fig. 4a), whereas differential analysis between ExICU rats with and without EV treatment (“D5EV vs D5”) identified a total of 109 DMs in lung tissues (Fig. 4b). Among which 68 DMs were overlapped between comparisons “D5 vs. D0” and “D5EV vs D5”, and 95% were with opposite direction of alteration, suggesting a restoring effect of EV treatment (Fig. 4c). Hierarchical clustering analysis of DMs showed distinct differences in lung metabolite profiles between group D5 and D0, validating the accuracy of differential analyses and confirming the strong impact of 5-day ICU condition on the metabolomic profiles in lung tissues. The metabolite profiles of lung between group D5EV and D5 were slightly distinct from each other (Fig. 4d).

According to the Metabolon annotation list, DMs were classified into nine categories, including Amino Acid, Cofactors and Vitamins, Carbohydrate, Energy, Lipid, Nucleotide, Partially characterized molecules, Peptides, and Xenobiotics. The majority DMs in lung belonged to the Lipid and Amino Acid category. Several metabolomic pathways were significantly upregulated in response to the 5-day ICU condition but were restored by EV treatment, for instance, “Fatty Acid Metabolism (Acyl Carnitine, Long Chain Saturated)”, “Fatty Acid, Dicarboxylate”, “Fatty Acid Metabolism (Acyl Choline)”, “Fatty Acid Metabolism (Acyl Carnitine, Polyunsaturated)”, “Fatty Acid, Dicarboxylate”, “Monoacylglycrol”, “Lysoplasmalogen” in the Lipid category; “Leucine, Isoleucine and Valine Metabolism” in the Amino Acid category; “Glycolysis, Gluconeogenesis, and Pyruvate Metabolism” in the Carbohydrate category; and “Purine Metabolism, (Hypo)Xanthine/Inosine containing” in the Nucleotide category; and “Benzoate Metabolism” in the Xenobiotics category but restored by EV treatment.

### BAL fluid proteomic signatures in EV treated and untreated animals exposed to 5 days mechanical ventilation

**Fig. 5:**
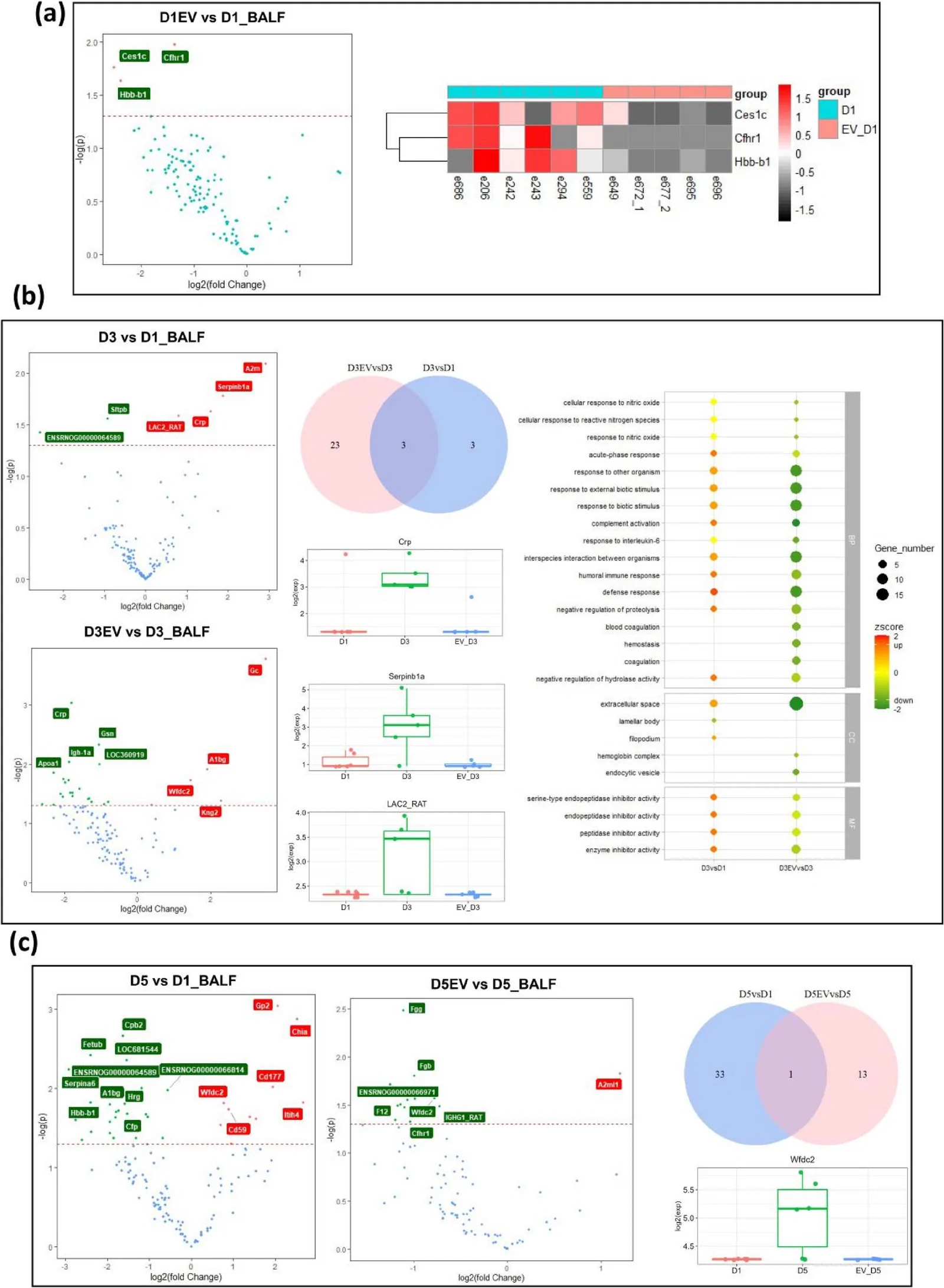
Proteomics of BAL fluid. An indicator of the restoring effects of EV treatment on lung tissue. Differentially expressed proteins (DEPs) in BAL fluid in ExICU rat with and without EV treatment at (**a**) day 1 (comparison “D1EV vs D1”), (**b**) day 3 (comparison “D3 vs D1” and “D3EV vs D3”), and (**c**) day 5 (comparison “D5 vs D1” and “D5EV vs D5”)

On the first day of EV treatment and mechanical ventilation, only 3 DEPs (Ces1C, Cfhr1, Hbb-b1) were identified in the BAL fluid when comparing treated vs. untreated animals (Fig. 5a). On the third day of EV treatment, 26 and 6 DEPs were identified in the comparisons “D3 vs. D1” and “D3EV vs. D3”, respectively. Crp, Serpinb1a, and LAC2_RAT overlapped between the two comparisons, which were upregulated in response to 3-day ICU condition but restored by EV treatment. According to GO enrichment analysis, the 26 DEPs identified in the comparison “D3 vs. D1” were mainly related to active immune responses, as reflected in the upregulation of GOBP terms “acute-phase response”, “response to external biotic stimulus”, “complement activation”, “humoral immune response”, and “defense response”. The 6 DEPs identified in the comparison “D3EV vs D3” were also related to these biological processes but with opposite direction of alteration, suggesting the restoring effect of EV treatment (Fig. 5b). On the fifth day of EV treatment, 34 and 14 DEPs were identified in the BAL fluid from comparison “D5 vs. D1” and comparison “D5EV vs. D5”, respectively. Wfdc2 was overlapped between two comparisons, which were up-regulated in response to 5-day ICU condition but restored by EV treatment (Fig. 5c).

### Diaphragm transcriptomics signatures in EV treated and untreated animals exposed to 5 days mechanical ventilation

**Fig. 6:**
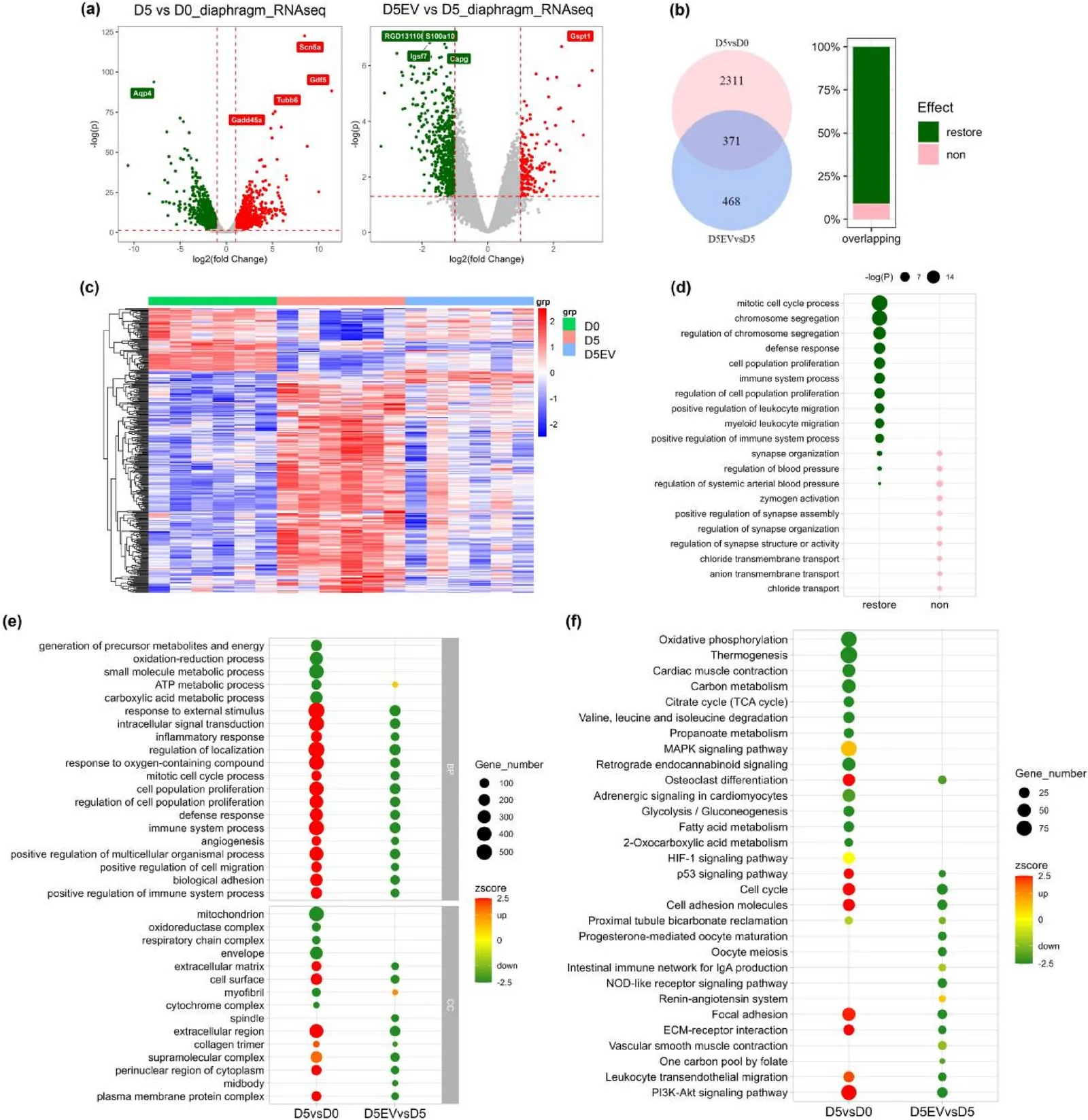
Transcriptomics reveal restoring effects of EV treatment on diaphragm tissue. (**a**) Volcano plots of differentially expressed genes (DEGs) in diaphragm tissues, i.e., the “D5 vs D0” and “D5EV vs D5” comparisons; (**b**) Overlapped DEGs between the comparisons “D5 vs D0” and “D5EV vs D5”; (**c**) Hierarchical clustering analysis of DEGs in diaphragm tissue of all groups; (**d**) KEGG terms enriched by the DEGs overlapped between comparisons “D5 vs D0” and “D5EV vs D5”; Comparative demonstration of enriched GO term (**e**) and KEGG term (**f**). D0: sham-operated rats (*n* = 6); D5: rats exposed to 5-day ICU condition (*n* = 6); D5EV: rats exposed to 5-day ICU condition and EV treatment (*n* = 6)

Compared with the sham-operated controls (D0), a total of 2,682 differentially expressed genes (DEGs) were identified in the diaphragm muscles of ExICU rats (D5), referred to as comparison “D5 vs D0” (Fig. 6a). Compared with the ExICU rats, a total of 839 differentially expressed genes (DEGs) were identified in the diaphragm muscles of ExICU rats with EV intervention (D5EV), referred to as comparison “D5EV vs. D5” (Fig. 6a). Among which, 371 DEGs were shared between the comparisons “D5 vs. D0” and “D5EV vs. D5”, and approximately 90% were with opposite direction of alteration, suggesting a restoring effect of EV treatment on the molecular alterations associated with VIDD (Fig. 6b). The restoring effect could be directly visualized by the gene expression pattern in hierarchical heatmap (Fig. 6c). Functional enrichment analysis revealed that the overlapped 371 DEGs were participating in biological processes like “immune system process”, “cell population proliferation”, and “synapse organization” (Fig. 6d).

The enriched GO (Fig. 6e) and KEGG (Fig. 6f) terms from each comparison were selected for comparative demonstration, respectively. The z-score was employed to predict the upregulated/activated or downregulated/inhibited status of the enriched terms. A stronger z-score reflects a more significant up- or down-regulation.

In the GO enrichment analyses, biological processes (BP) and cellular components (CC) related to metabolism and energy production in diaphragm muscles were severely impaired after the 5-day ICU condition, as reflected by the downregulation or inhibited status (negative z-scores) of the GOBP terms “generation of precursor metabolites and energy”, “ATP metabolic process”, as well as the GOCC terms “mitochondrion”, “respiratory chain complex”. KEGG enrichment analyses revealed inactivation (negative z-scores) of signaling pathways participating in the energy production after the 5-day ICU conditions, including “oxidative phosphorylation”, and “Citrate cycle (TCA cycle)”. Metabolism was also significantly impaired, as reflected by the negative scores of KEGG terms like “Carbon metabolism, “Valine, Leucine and Isoleucine Degradation”, “Fatty Acid Metabolism”, and “Glycolysis/Gluconeogenesis”, etc. However, the impaired metabolism and energy production was not restored by EV treatment.

On the other hand, several biological processes and signaling pathways related to immune and inflammatory activities were significantly upregulated after the 5-day ICU condition, as reflected by the positive z-scores of the GOBP terms “response to external stimulus”, “inflammatory response”, “defense response”. “immune system response”, and the KEGG terms “leukocyte transendothelial migration”, “p53 signaling pathway”, etc. The activated immune and inflammatory responses were partially restored by EV treatment. The ameliorating effect of EV treatment was also observed in genes involved in GOBP terms “cell population proliferation”, “angiogenesis”, GOCC terms “extracellular matrix”, and KEGG terms “cell cycle”, “cell adhesion molecules”, “Focal adhesion”, “ECM-receptor interaction”, and “PI3K-Akt signaling pathway”.

**Fig. 7:**
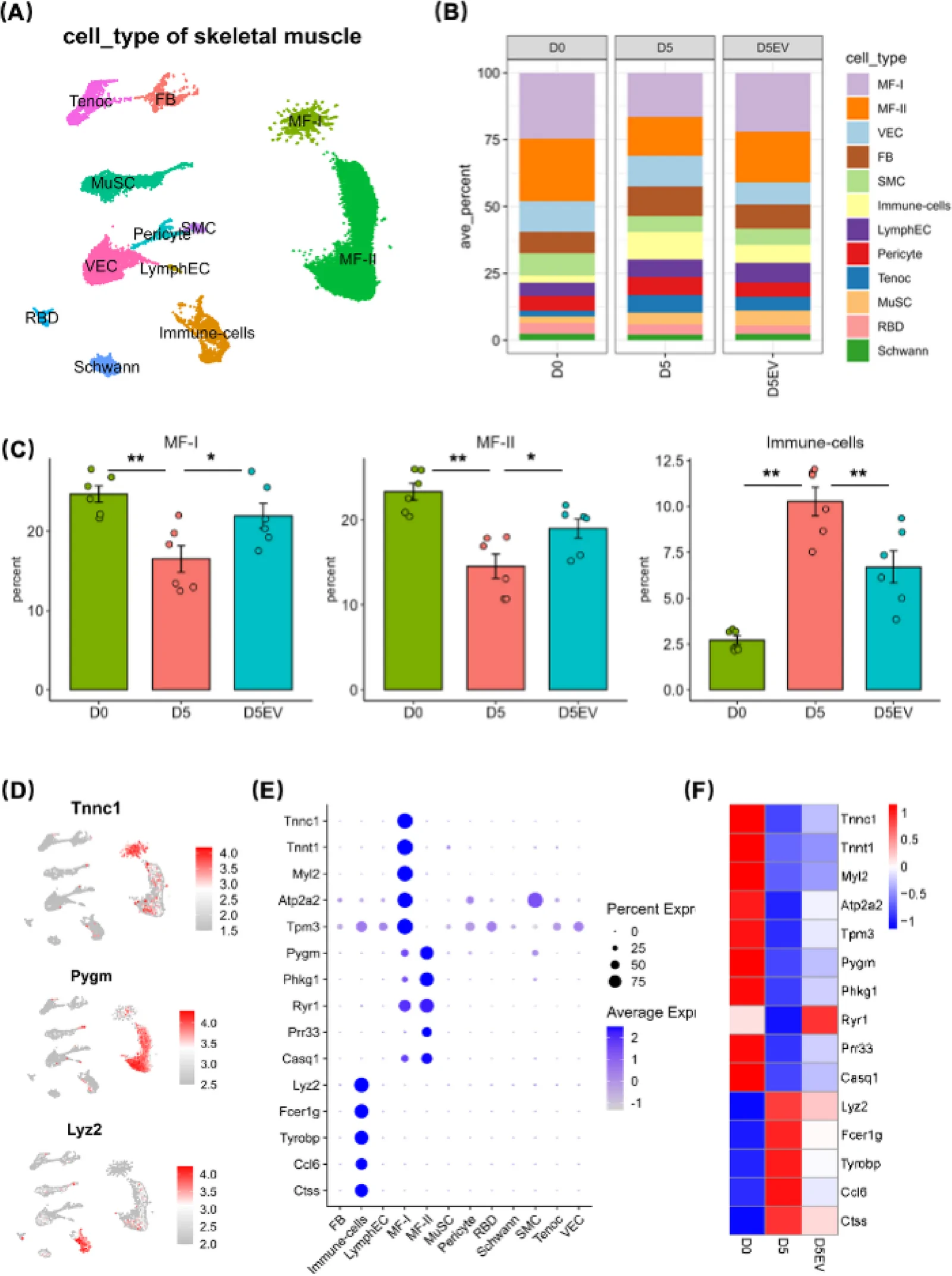
Estimation of cell type composition of diaphragm muscle in each group. (**A**) t-SNE presentation of a single-cell reference atlas of healthy diaphragm muscle, based on previously published single-cell sequencing datasets (Mouse). Cells were categorized into 12 primary clusters, each was presented by a different color: type I, slow-twitch myofibers (MF-I); type II, fast-twitch myofibers (MF-II), fibroblast (FB), muscle stem cells (MuSC), vascular endothelial cells (VEC), tenocytes (Tenoc), Immune-cells, lymphatic endothelial cells (LymphEC), red blood cell debris (RBD), smooth muscle cells (SMC), Pericytes, Schwann cells. (**B**-**C**) Estimation of cell type composition and their relative abundance in diaphram muscle from group D0, D5 and D5EV by cellular deconvolution analysis. (**D**-**E**) Confirmation of specificity of the top 5 marker genes of MF-I, MF-II and immune-cells by t-SNE and gene marker dot, respectively. (**F**) Expression level of the top 5 marker genes for MF-I (Tnnc1, Tnnt1, Myl2, Atp2a2 and Tpm3), MF-II (Pygm, Phkg1, Ryr1, Prr33 and Casq1) and immune-cells (Lyz2, Fcer1g, Tyrobp, Ccl6 and Ctss) in each group. * indicates p < 0.05, ** indicates p < 0.01.

Based on published single-cell sequencing datasets, we predicted a comprehensive single-cell atlas of diaphragm tissue comprising 12 distinct cell populations. These included including slow-twitch type I myofibers (MF-I); fast-twitch type II myofibers (MF-II), fibroblasts (FB), muscle stem cells (MuSC), vascular endothelial cells (VEC), tenocytes (Tenoc), Immune-cells, lymphatic endothelial cells (LymphEC), red blood cell debris (RBD), smooth muscle cells (SMC), Pericytes, Schwann cells (Fig. 7a-b).

Cellular deconvolution analysis integrating our bulk RNAseq data from each group (D0, D5 and D5EV) revealed that both slow-twitch (Type I) and fast-twitch (Type II) muscle fibers in the diaphragm significantly decreased in response to mechanical ventilation. Notably, EV treatment significantly reversed these decreases in both muscle fiber types (Fig. 7c), a finding further validated by expression analysis of the top 5 marker genes for Type I (Tnnc1, Tnnt1, Myl2, Atp2a2 and Tpm3) and type II muscle fibers (Pygm, Phkg1, Ryr1, Prr33 and Casq1; Fig. 7d-f).

Concomitantly, diaphragm tissue from the D5 group exhibited a significant increase in immune cell infiltration compared to the sham-operated controls (D0). EV treatment attenuated this inflammatory response by promoting muscle repair and regeneration through satellite cell activation (Fig. 7c), as confirmed by expression analysis of the top 5 marker genes of immune cell (Lyz2, Fcer1g, Tyrobp, Ccl6 and Ctss; Fig. 7d-f). Similar restorative effects of EV treatment were also observed in pericytes, fibroblasts, and tenocytes (Supplementary Fig. 2), suggesting a multifaceted therapeutic mechanism.

### Diaphragm metabolomics signatures in EV treated and untreated animals exposed to 5 days mechanical ventilation

**Fig. 8:**
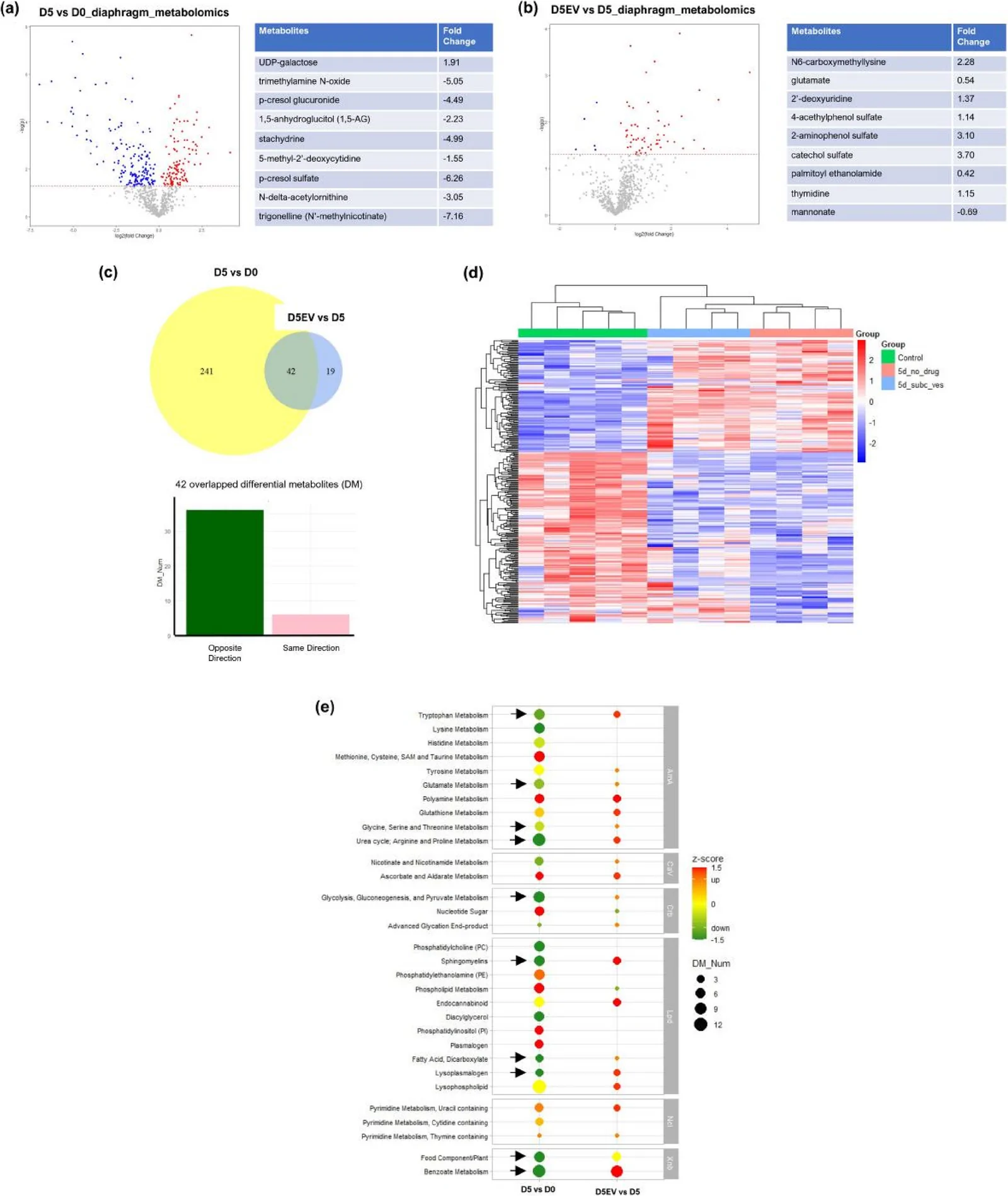
Metabolomics reveal restoring effects of EV treatment on diaphragm tissue. Differential metabolites (DMs) identified in “D5 vs D0” (**a**) and “D5EV vs D5” (**b**); (**c**) Overlapped DMs between comparisons “D5 vs D0” and “D5EV vs D5” and percentage of DMs with restoring effect of EV treatment; (**d**) Hierarchical clustering analysis of DMs in lung tissue of all groups; (**e**) Comparative demonstration of enriched metabolic pathways. D0: sham-operated rats (*n* = 5); D5: rats exposed to 5-day ICU condition (*n* = 4); D5EV: rats exposed to 5-day ICU condition and EV treatment (*n* = 5)

Differential analysis between 5-day ventilated and sham-operated controls (comparison “D5 vs D0”) demonstrate a total of 283 differential metabolites (DMs) in diaphragm muscles (Fig. 8a), whereas differential analysis between 5-day ventilated rats with and without EV treatment (“D5EV vs. D5”) identified a total of 61 DMs in diaphragm muscles (Fig. 8b). Among which 42 DMs were overlapped between comparisons “D5 vs. D0” and “D5EV vs. D5”, and 95% were with opposite direction of alteration, suggesting a restoring effect of EV treatment (Fig. 8c). Hierarchical clustering analysis of DMs showed distinct differences in metabolite profiles of diaphragm between group D5 and D0, validating the accuracy of differential analyses and confirming the strong impact of the 5-day ICU condition on metabolomic profiles in diaphragms (Fig. 8d).

The majority of DMs in diaphragm belonged to the Lipid and Amino Acid category. Several metabolomic pathways were significantly downregulated in response to the 5-day ICU condition but were restored by EV treatment, for instance, “Sphingomyelins”, “Fatty Acid, Dicarboxylate”, and “Lysoplasmalogen” in the Lipid category; “Tryptophan Metabolism”, “Glutamate Metabolism”, “Glycine, Serine and Threonine Metabolism”, and “Urea cycle; Arginine and Proline Metabolism” in the Amino Acid category; “Glycolysis, Gluconeogenesis, and Pyruvate Metabolism” in the Carbohydrate category; and “Benzoate Metabolism” in the Xenobiotics category (Fig. 8e).

## Discussion

The major findings from this study were: (1) An approximately 50% decline in diaphragm muscle fiber area and specific force in response to the 5-day ICU condition (controlled mechanical ventilation and immobilization), (2) The 5-day mechanical ventilation induced significant lung alveolar pathology with inflammation, edema and ectasia, as well as increased interstitial and peribronchial inflammation, (3) Lung genomic analyses revealed changes related to injury, such as wound healing and activation of immune responses in accordance with histopathology. Metabolomic analyses revealed that the majority of the differentially expressed metabolites belonged to the lipid and amino acid pathways, such as fatty acid metabolism and the “Leucine, Isoleucine and Valine Metabolism”, but also “Glycolysis, Gluconeogenesis, and Pyruvate Metabolism” in the Carbohydrate category. (4) In accordance with lung pathology and genomics, BAL fluid proteomics revealed the release of proteins from the lungs involved in the activation of immune responses after 5 days controlled mechanical ventilation. (5) Diaphragm genomic analyses revealed upregulation of signaling pathways related to immune and inflammatory activities. The majority of the differentially expressed metabolites in the diaphragm belonged to the Lipid and Amino Acid category which were downregulated in response to the 5-day ICU condition. (6) Systemic treatment with EVs derived from human BM-MSC had a restoring effect on the immune response observed in lung, BAL fluid and diaphragm muscle as well as on lung pathology and diaphragm muscle fiber size/function. In addition, the EV treatment promoted muscle repair and regeneration by activating muscle satellite cells. (7) Downregulation of both fast and slow myosin and myosin associated proteins were reversed by the EV treatment suggesting the importance of the inflammatory response in regulating myosin expression.

To our knowledge, this is the first time BM-MSC-derived EVs have been shown to reduce the diaphragm dysfunction (VIDD) and ventilator induced lung injury (VILI) associated with modern critical care as well as combatting metabolic changes in lungs and respiratory muscles. BM-MSCs are immune modulatory cells with angiogenetic, anti-apoptotic and anti-fibrotic properties [26]. These cells are clinically accepted, can be harvested from healthy donors, and can be used in allogeneic settings for treatment of various systemic inflammatory conditions including [27–29]. The safe profile of BM-MSCs together with their immune modulatory characteristics make these cells a potential biological drug aiming at reducing the negative consequences of long-term ICU treatment on lung and respiratory muscle. The effect of BM-MSCs is to a large extent mediated by secreted EVs [30]. EVs have the advantage over BM-MSCs that they can be stored for long periods of time at – 20 °C (or even for some days refrigerated at + 4 °C) without losing their efficacy, making EVs main candidates for replacement of cumbersome BM-MSC therapies. In the present study, this is supported in the rat which survived the BM-MSC treatment for 5 days without adverse effects, had similar restoring effects on diaphragm muscle size and function as in the EV treated animals. The reduced alveolar damage and anti-inflammatory effects observed in the current experimental ICU model are in accordance with previous experimental and clinical studies (see [27]). The reduced VILI and VIDD together with the anti-inflammatory effects on lung and BAL fluid by the EV treatment suggest a potential pathophysiological link between VILI and VIDD. Thus, cytotoxic factors released from the highly vascularized injured lung may impact negatively on the diaphragm. In line with this, a pathophysiological link has been forwarded between the release of cytotoxic hyperphosphorylated tau from injured lungs and the associated brain injury [31]. However, this needs further scientific attention, we are currently exploring how EVs target the diaphragm directly or indirectly via the lung and the release of specific cytotoxic factors.

Intravenous BM-MSC treatment has been shown to be safe in clinical trials in individuals with aging-related frailty [32]. This is of specific interest in this context, since old age and muscle wasting are the two factors which most strongly predict mortality and morbidity in mechanically ventilated ICU patients (see [33]). In these studies, it was sufficient to give BM-MSCs as a single dose, but this needs not be true for EV treatment where multiple doses may be needed. The more pronounced restoring effect by EV treatment on the BAL fluid proteomic signature on the third than the fifth day after EV administration supports multiple EV administrations. In control experiments, secretion from salivary glands and a mild inflammatory response is typically observed around the tracheal tube after 1st or 2nd day of mechanical ventilation, but this reaction was not observed in the BM-MSC treated rat. A similar inhibition of this inflammatory response was observed in the EV treated animals in the first 3–4 days of treatment, but a mild inflammatory response around the tracheal tube was observed on day 4 and 5, lending support to the benefit of multiple EV administrations. Intravenous administration of drugs, fluids and nutrition is routine in sedated and mechanically ventilated ICU patients and repeated intravenous administrations of EV does not represent a logistic problem and may prove advantageous in the clinical setting. Furthermore, EV treatment has significant advantages over BM-MSC treatment related to lower costs, simpler storage and logistics compared to cell treatment.

### Study limitations

The relatively small number of animals included in this study represents a limitation of the study. The small number of observations is due to the costly, demanding and complex experimental model and methods for analyses. To our knowledge, this is the only murine model allowing long-term studies in an immobilized and mechanically rat mimicking the ICU condition including 24 h/d monitoring of the rat on site. However, the model has proved invaluable in the investigation of the limb and respiratory muscle dysfunction associated with long-term exposure to the ICU condition (immobilization and mechanical ventilation) in the absence of an underlying disease (unethical in a clinical study). Despite the relatively small number of animals studied, the effects of the ICU condition on lung and diaphragm are profound as well as the impact of EV treatment.

The absence of an appropriate rat single-cell reference dataset for cell-type deconvolution of rat diaphragm tissue may introduce uncertainty due to interspecies differences. However, given that only mouse and human single-cell datasets were available, we chose the mouse dataset for the analysis since we believe it provides a more biologically appropriate reference for cross-species deconvolution.

The lack of a reference group treated with the vehicle without EVs represents an additional limitations which will be rectified in future experiments.

## Conclusion

Treatment with EVs derived from BM-MSCs significantly recovered genomic, proteomic and metabolomic changes in lungs, BAL fluid and diaphragm as well as significantly mitigated the ventilator induced diaphragm muscle fiber atrophy and specific force loss induced by the lifesaving intervention of long-term mechanical ventilation frequently used in modern critical care. Thus, EV treatment is forwarded as a potent intervention aimed at shortening the weaning process in long-term mechanically ventilated ICU patients to reduce duration of ICU stay and risk of secondary complications associated with prolonged mechanical ventilation such as pneumonia and the increased risk of morbidity and mortality.

## Supplementary Information

Supplementary Material 1.

Supplementary Material 2.

## Data Availability

The datasets used during the current study are available from the corresponding author on a reasonable request.
