# Supplementary material for: Combatting ventilator induced diaphragm dysfunction with human bone marrow mesenchymal stromal cell-derived extracellular vesicles
Source: Skelet Muscle. 2026 Jun 13;16:32. doi: 10.1186/s13395-026-00433-6 (PMC13495324; doi:10.1186/s13395-026-00433-6)
Supplement: Supplementary file 1 [file 13395_2026_433_MOESM1_ESM.docx]

**Supplementary Figure Legends**

**Supplementary Figure 1.** **Characterization of the BM-MSC derived EVs.** (a) Nanoparticle tracking analysis (NanoSight 3000) of the EVs. (b) Transmission electron microscopy of the EVs. Scale bar, 100 nm. (c) Western-blot analysis of the EVs (EV) and the BM-MSC cell lysates (Cell lysate) for surface markers of EVs CD63 and CD81; for internal marker of EVs TSG101; and for negative marker of EVs Calnexin.

**Supplementary Figure 2. Estimation of cell type composition and their relative abundance in diaphragm muscle from group D0, D5 and D5EV by cellular deconvolution analysis.** type I, slow-twitch myofibers (MF-I); type II, fast-twitch myofibers (MF-II), fibroblasts (FB), muscle stem cells (MuSC), vascular endothelial cells (VEC), tenocytes (Tenoc), Immune-cells, lymphatic endothelial cells (LymphEC), red blood cell debris (RBD), smooth muscle cells (SMC), Pericytes, Schwann cells. * indicates p < 0.05, ** indicates p < 0.01.

**Supplementary Figure 1**

**Supplementary Figure 2**
